# The placenta at term: insights from the Loke Centre for Trophoblast Research 18th Annual Meeting, 2025

**DOI:** 10.1242/bio.062284

**Published:** 2026-01-22

**Authors:** Noa Hasky

**Affiliations:** ^1^ Department of Obstetrics and Gynaecology, University of Cambridge, NIHR Cambridge Comprehensive Biomedical Research Centre, Cambridge CB2 0SW, UK; ^2^ Loke Centre for Trophoblast Research, Department of Physiology, Development and Neuroscience, University of Cambridge, Cambridge CB2 3EL, UK

**Keywords:** Preeclampsia, Fetal growth restriction, Gestational diabetes

## Abstract

The Loke Centre for Trophoblast Research Annual Meeting, ‘The Placenta at Term’, was held on 7-8 July, 2025, at the University of Cambridge, UK. The meeting brought together leading clinical and basic scientists from around the world to explore how robust research methods can improve understanding of placental complications such as preeclampsia, fetal growth restriction, and gestational diabetes. This Meeting Review highlights emerging research directions and emphasises the remarkable potential of the placenta, not only as a window into obstetrical complications, but also as a diagnostic tool for predicting the short- and long-term health of both mother and child.

## Teaser

**Summary:** Emerging research positions the placenta as a unique window into obstetrical complications and a determinant of lifelong health, opening new opportunities for prediction, prevention and intervention.

## Introduction

Placental complications are a leading cause of maternal and neonatal morbidity and mortality worldwide (Cresswell et al., 2025; Lawn et al., 2023), yet the underlying mechanisms remain elusive. Consequently, management of severe cases often depends on induction of labour, forcing clinicians to weigh the risks of disease progression against those of prematurity, with profound health and economic consequences (Stevens et al., 2017). To make meaningful progress, the field must move beyond symptomatic management and toward a deeper mechanistic understanding of pathophysiology. Advanced *in vitro* models such as stem cell-derived trophoblast lines, 3D organoids, and placenta-on-a-chip platforms are providing powerful tools to explore the molecular processes that underpin placental development (Aye, 2024). However, they cannot fully capture the complexity of fetal-placental-maternal interactions, which require *in vivo* studies and remain the essential link to clinical translation.

The placenta exemplifies this opportunity through its unique nature: originating from the embryo while shaped by maternal physiology, it acts as the hub of bidirectional communication between mother and fetus, driving key adaptations that determine pregnancy and lifelong outcomes. Thus, its value as a research subject lies not only in the potential to uncover the causes of pregnancy complications, but also in offering a window for predicting long-term health outcomes in both mother and child. Yet despite this promise and its ready accessibility at delivery, the placenta remains underutilised as a diagnostic tool and underrepresented in large-scale biological research (Bhattacharya et al., 2022). The Loke Centre for Trophoblast Research (Loke CTR) 2025 Annual Meeting, placed this paradox at the forefront, uniting clinical and basic scientists to explore how to harness the diagnostic and research potential of the placenta.

**Fig. 1.**
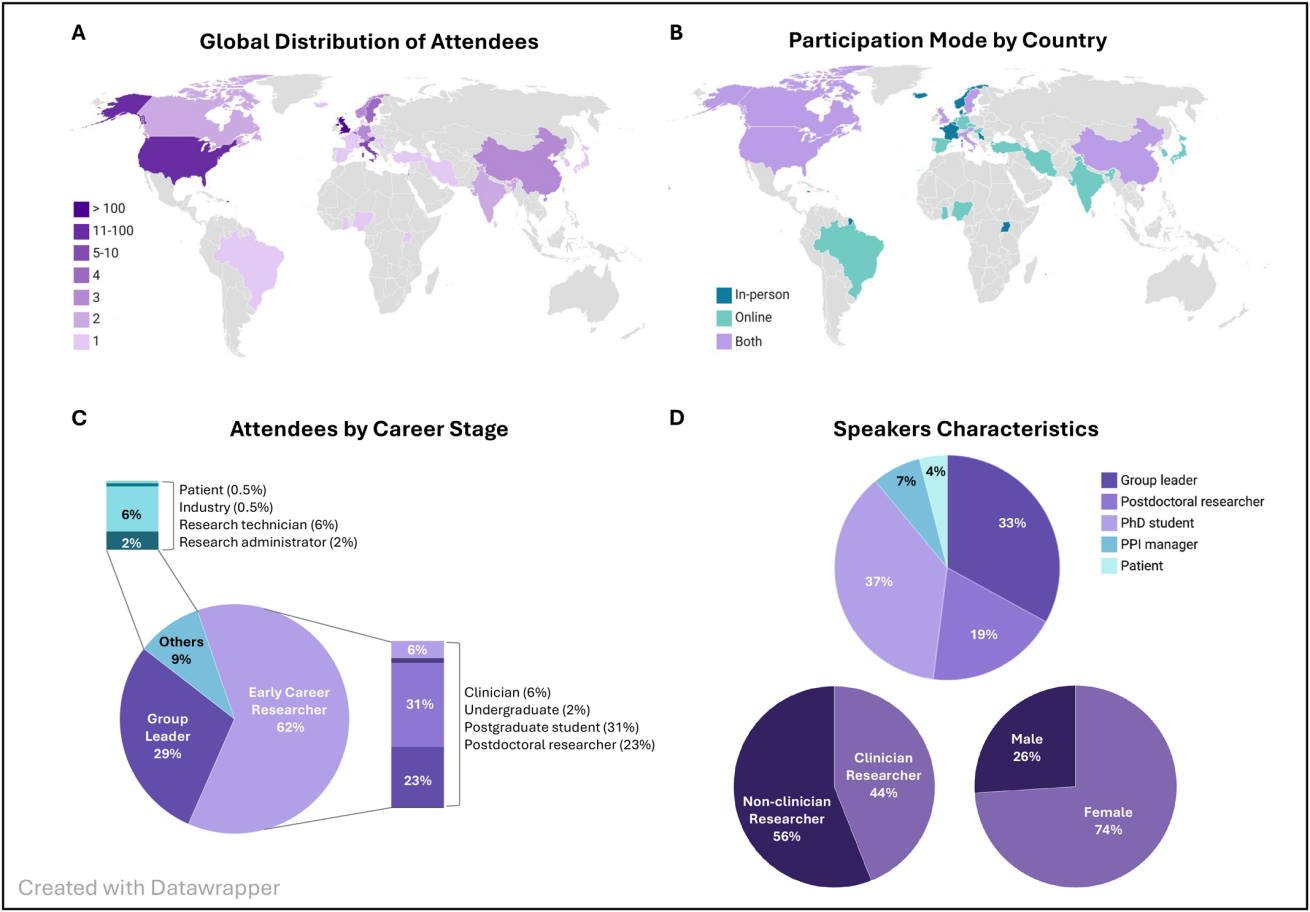
**Overview of annual meeting participation.** (A) Global distribution of attendees by country of institutional affiliation (absolute numbers). (B) Participation mode by country of origin (in-person: dark teal; online: light teal; both: light purple). (C) Attendee composition by career stage. (D) Speaker characteristics by career stage, gender, and clinical background.

The meeting attracted participants from across the globe, welcoming 249 attendees from 30 countries (Fig. 1A). To accommodate growing international interest, it was held in a hybrid format, with 176 participants attending in person (71%) and 73 online (29%), extending representation to 14 additional countries (Fig. 1B). Early-career researchers (ECRs) made up more than half of all participants, highlighting both the vitality of the field and Loke CTR's commitment to supporting the next generation of placental scientists (Fig. 1C). This commitment was further reflected in the program: 41 posters were presented, the vast majority by ECRs (36; 88%), with 15 selected for flash talks. The meeting also featured eight keynote lectures from group leaders and three from patient and public involvement and engagement (PPIE) delegates, underscoring the importance of embedding patient perspectives in research.

Diversity was also reflected among the 27 invited speakers, 74% of whom were women, with a balance between non-clinical (56%) and clinical researchers (44%) (Fig. 1D), reflecting the Loke CTR's goal to promote collaboration across disciplines and bridge basic and clinical placental research.

Overall, the program created a forum that cultivated inclusivity and collaboration across career stages, while showcasing how bottom-up approaches are advancing placental research. The following sections review the major scientific themes, from new models of placental pathophysiology to innovative technologies, maternal-placental crosstalk, and placental-guided prevention strategies.

## New frontiers in understanding placental pathophysiology

Preeclampsia is a serious complication affecting 2-4% of pregnancies and causing ∼70,000 maternal and 500,000 perinatal deaths annually (Dimitriadis et al., 2023). Accumulating evidence has led clinicians and placental researchers to classify preeclampsia according to disease onset, reflecting potentially different underlying origins. Early-onset preeclampsia (EOPE; diagnosed before 34 weeks' gestation) is attributed to impaired placentation and is most often accompanied by fetal growth restriction (FGR), while late-onset preeclampsia (LOPE; diagnosed at or after 34 weeks' gestation) is more commonly associated with maternal risk factors. Prof. Annetine Staff (University of Oslo, Norway) proposed a common pathway for both forms, identifying syncytiotrophoblast stress as the central mediator towards clinical manifestations.

Staff's presentation was delivered as a tribute to Prof. Chris Redman and centred on their jointly developed ‘Twilight Placenta’ concept. This model suggests that as pregnancy advances towards term and post-term, the placental villi become overcrowded, reducing placental perfusion and oxygen uptake (Serov et al., 2015), similar to hypoperfusion demonstrated in placentas of EOPE (Sohlberg et al., 2014). In fact, the twilight placenta shares several features with EOPE placentas, including enhanced oxidative stress (Burton et al., 2021), endoplasmic reticulum stress (Yung et al., 2008), increased senescence markers (Cindrova-Davies et al., 2018), and common histopathological findings (Calvert et al., 2013; Carroll et al., 2022). Maternal blood from post-term and EOPE pregnancies contains more fetal-origin cells (Levine et al., 2004) and shows an altered placental-secreted angiogenic profile: lower placental growth factor (PLGF), higher soluble fms-like tyrosine kinase-1 (sFlt-1), and an elevated sFlt-1/PLGF ratio, potentially reflecting increased syncytiotrophoblast cellular stress (Mitlid-Mork et al., 2020). In this framework, Staff suggests that EOPE results from extrinsic uteroplacental malperfusion caused by defective spiral artery remodelling, whereas LOPE arises from intrinsic intraplacental malperfusion of a condensed and ageing twilight placenta, with both pathways culminating in syncytiotrophoblast stress, leading to preeclampsia (Redman and Staff, 2015).

Another perspective on the development of LOPE was introduced by Prof. Ananth Karumanchi (Cedars-Sinai Medical Center, Los Angeles, USA), whose pioneering work identifying the role of circulating angiogenic factors in preeclampsia, has fundamentally advanced our understanding of the disease and paved the way for biomarker-guided diagnosis (Levine et al., 2004). Prompted by the observation that women with reduced renal reserve (e.g. single kidney after donation) are at increased risk of LOPE, despite having normal kidney function prior to pregnancy, Karumanchi's team developed a uni-nephrectomy mouse model to investigate the mechanism. In these mice, reduced renal reserve blunted the physiological glomerular hyperfiltration required to sustain the expansion of maternal plasma volume, which led to placental malperfusion and a preeclampsia-like phenotype. Complementing the ‘Twilight Placenta’ model, this work suggests that maternal limitations in physiological adaptation can also produce preeclampsia phenotype through the common pathway of placental malperfusion. Administering L-kynurenine, which was reduced in the blood of uni-nephrectomised mice compared with controls, rescued them from the preeclampsia-like phenotype (Dupont et al., 2022) and investigations into the underlying mechanism are ongoing.

## Harnessing innovative technologies for placental research

A central theme of the meeting was the application of high-throughput technologies to uncover altered pathways in placental dysfunction, offering new clues to disease mechanisms and potential diagnostic markers. Prof. Gordon Smith (University of Cambridge, UK) leads the Pregnancy Outcome Prediction Studies (POPS1, 2008-2013; and POPS2, 2020-2026); large, prospective cohorts comprising over 9000 nulliparous singleton pregnancies. These cohorts are deeply characterised through extensive clinical, genetic, sonographic, and biological sampling from pregnancy through birth (Gaccioli et al., 2017). Building on this robust resource, Smith's team has produced influential insights across genomics (Mack et al., 2024), transcriptomics (Gong et al., 2021), and metabolomics (Sovio et al., 2020), and is now pioneering proximity extension assay (PEA) proteomics to identify novel predictors and mechanistic markers for obstetrical complications. This method uses pairs of antibodies tagged with complementary DNA strands; when both bind their target protein, the strands hybridise, forming a unique DNA sequence that can be amplified and quantified, enabling measurement of ∼3000 proteins from just 6 μl of serum.

Applying mathematical modelling and gene-enrichment analysis, the team identified distinct protein expression patterns in preeclampsia and FGR. In early pregnancy, protein changes in both conditions were strongly correlated, pointing to shared pathways. Later, the profiles diverged, preeclampsia showed angiogenesis-related changes and an earlier shift to protein patterns typical of term, consistent with the twilight placenta concept. This integrative approach could disentangle shared versus disease-specific mechanisms and deliver translatable biomarkers for early detection.

Extending innovation to placental structure, Prof. Alexander Heazell (University of Manchester, UK) applies advanced imaging and computational modelling to investigate the origins of stillbirth and placental dysfunction. His group applies whole-slide imaging and digital quantification of placental structural and cellular features, reducing the subjectivity of conventional histology (Ptacek et al., 2016). A complementary placental explant model replicates pathological conditions *in vitro*, with findings validated against quantified *in vivo* pathology (Baker et al., 2021). Recently, Heazell introduced Synchrotron CT imaging with machine-learning analysis to generate high-resolution 3D reconstructions of villous and microvascular networks. Building on these maps, his team feeds morphometric outputs (e.g. branch length, surface area) into oxygen-transport models and ultimately aims to link them to MRI T2*, a readout of tissue oxygenation, to develop a non-invasive approach for detecting impaired placental perfusion during pregnancy.

## Insights into maternal-placental crosstalk

Diabetes affects 16% of live births worldwide (around 23 million each year) and can have lasting consequences for both mother and child (Gestational Diabetes - International Diabetes Federation). To explore ways of mitigating these effects, Prof. Karen Forbes (University of Leeds, UK) presented an integrative approach to studying maternal-placental crosstalk in gestational diabetes mellitus (GDM). Extracellular vesicles (EVs) secreted by the placenta were shown to influence maternal physiology in GDM (James-Allan et al., 2020). Forbes showed that EVs can travel both ways, with GDM-altered maternal EVs reaching the placenta and reprogramming its structure and function (Farrelly et al., 2023). In her studies, EVs isolated from GDM patients with large-for-gestational-age (LGA) infants carried altered microRNA and glycoprotein content (Kennedy et al., 2019). When fluorescently labelled and injected into pregnant mice, EVs localised to the placenta, causing structural changes, altered metabolism and increased fetal weight (Quilang et al., 2022b).

Forbes focused on miRNA-375, produced under a pancreas-specific promoter, which was elevated in EVs from GDM-LGA cases. Her team exposed human pancreatic islets to mild hyperglycaemia, isolated the resulting EVs, and showed that placental explants internalised them, raising miRNA-375 levels. Injecting these pancreatic-derived EVs into pregnant mice replicated the placental and fetal changes seen with GDM-derived EVs (Quilang et al., 2022a). This aligns with recent evidence that maternal adipose-derived EVs can regulate placental function and fetal growth in GDM (Jayabalan et al., 2025). Together, these findings support a model in which hyperglycaemia alters maternal EVs, reprogramming the placenta and driving vascular and metabolic changes that promote LGA outcomes.

Building on the concept of bidirectional maternal-placental communication, Prof. Perrie O'Tierney-Ginn (Tufts University, USA) investigates how the placenta modulates maternal metabolism to influence fetal growth. Her work focuses on late gestation, a critical window when physiological insulin resistance and hyperlipidaemia maximise nutrient delivery to the fetus. Central to this is the role of placental chromosome 19 microRNA cluster (C19MC), secreted into maternal circulation throughout pregnancy with the highest circulating levels in late pregnancy. Early pregnancy circulating levels of one such microRNA, miR-517a-3p, predict greater maternal insulin resistance and higher neonatal adiposity at term (Alvarado-Flores et al., 2025), while the placental level of another, miR-3940-3p is associated with increased maternal insulin resistance at time of birth (Alvarado-Flores et al., 2021).

Unpublished *in-vitro* studies show that synthetic C19MC reduce insulin-stimulated glucose uptake in human muscle cells, implicating the placenta in reprogramming maternal metabolism and promoting fetal fat accrual. This positions miRNA-mediated placental signalling as a key metabolic regulator, complementing direct nutrient transfer and highlighting crosstalk as a potential intervention target to prevent excessive offspring adiposity.

## Placental-guided prevention strategies

Prof. David Williams (University College London, UK) specialises in the management of pregnant women with complex medical and placental disorders. He illustrated how detailed placental pathology can help unravel disease mechanisms and guide targeted preventive strategies. His focus was chronic histiocytic intervillositis (CHI)-a rare, non-infectious placental disorder (∼6:10,000 placentas) linked to recurrent miscarriage, stillbirth, and FGR, often escalating in subsequent pregnancies (Contro et al., 2010). Histologically, CHI is marked by dense maternal immune cell infiltration (CD68^+^ macrophages, cytotoxic T cells) and complement deposition within the intervillous space (Bos et al., 2018). This immune signature closely mirrors the histological complexes seen in solid-organ transplant rejection (Brady et al., 2021). Combined with the threefold higher prevalence of autoimmune disease (Cornish et al., 2022) and improved outcomes with surrogacy (Cornish et al., 2023), these findings support CHI as a form of alloimmune-mediated placental rejection.

Building on this insight, Williams and colleagues trialled a targeted immunomodulatory regimen in a cohort of 20 women with recurrent CHI. Treatment consisted of anti-rejection therapy (ART) administered from pre-conception until childbirth, alongside low-dose aspirin. This approach markedly improved obstetric outcomes, reflected in a significant increase in live birth rates (unpublished). Analysis of treatment failures highlighted the importance of initiating therapy before conception and closely monitoring for drug toxicity. Ongoing mechanistic studies are investigating maternal-fetal HLA mismatch (Brady et al., 2024) and anti-partner HLA antibodies (Benachi et al., 2021) as potential mediators of CHI-related alloimmunity, potentially paving the way for new targeted preventive strategies.

Extending the concept of placental indicators as predictive tools, Prof. Catherine Aiken (University of Cambridge, UK) has demonstrated that antenatal markers of placental dysfunction can predict long-term educational attainment. Leveraging the POPS cohort, she linked placental dysfunction cases, defined by ultrasound/biochemical markers, to the UK National Pupil Database, allowing large-scale analysis with reduced retention bias.

Aiken's analysis revealed that term-born children diagnosed *in utero* with FGR were ∼50% more likely to underperform in reading, writing, and mathematics at ages 5-7. Notably, one in five failed the age-6 phonics screen, and these associations were consistent whether FGR was defined by ultrasound or biochemical criteria (Olga et al., 2023). The double-blinded POPS design enabled stratification into clinically detected and undetected FGR, with both groups showing similar educational outcomes, implicating the natural history of placental insufficiency, rather than obstetric management, as the key driver. Since early academic assessments are well-established predictors of later performance (Duncan et al., 2007), this integration of rigorous antenatal phenotyping with national record offers a powerful framework for identifying at-risk children and guiding timely, targeted interventions.

## Nothing about us without us

This session emphasised how central PPIE is in ensuring that placental research truly reflects the needs and experiences of those affected, thereby enhancing its impact. Sarah Falkland, a nurse and founder of Mission Preeclampsia, shared her personal journey as a preeclampsia survivor turned advocate. She emphasised the deep and lasting physical and emotional toll the condition can cause well beyond childbirth, calling for urgent action through greater awareness, effective prevention, and ongoing follow-up support. In this context, the POPPY trial (preconception to post-partum study of cardiometabolic health in primigravid pregnancy) provided a platform for demonstrating how structured PPIE can influence research. As part of the trial, Ewa Czekaj, project manager and PPIE lead (University of Cambridge, UK), and Rachel Ibikunle, research midwife and PPIE lead (Imperial College London, UK), showed how a diverse PPIE panel can shape study design, recruitment, and dissemination. Their efforts resulted in broader public outreach through survivor involvement in press releases, community events, and online storytelling.

## Conclusions: shaping the future of placental research

**Fig. 2.**
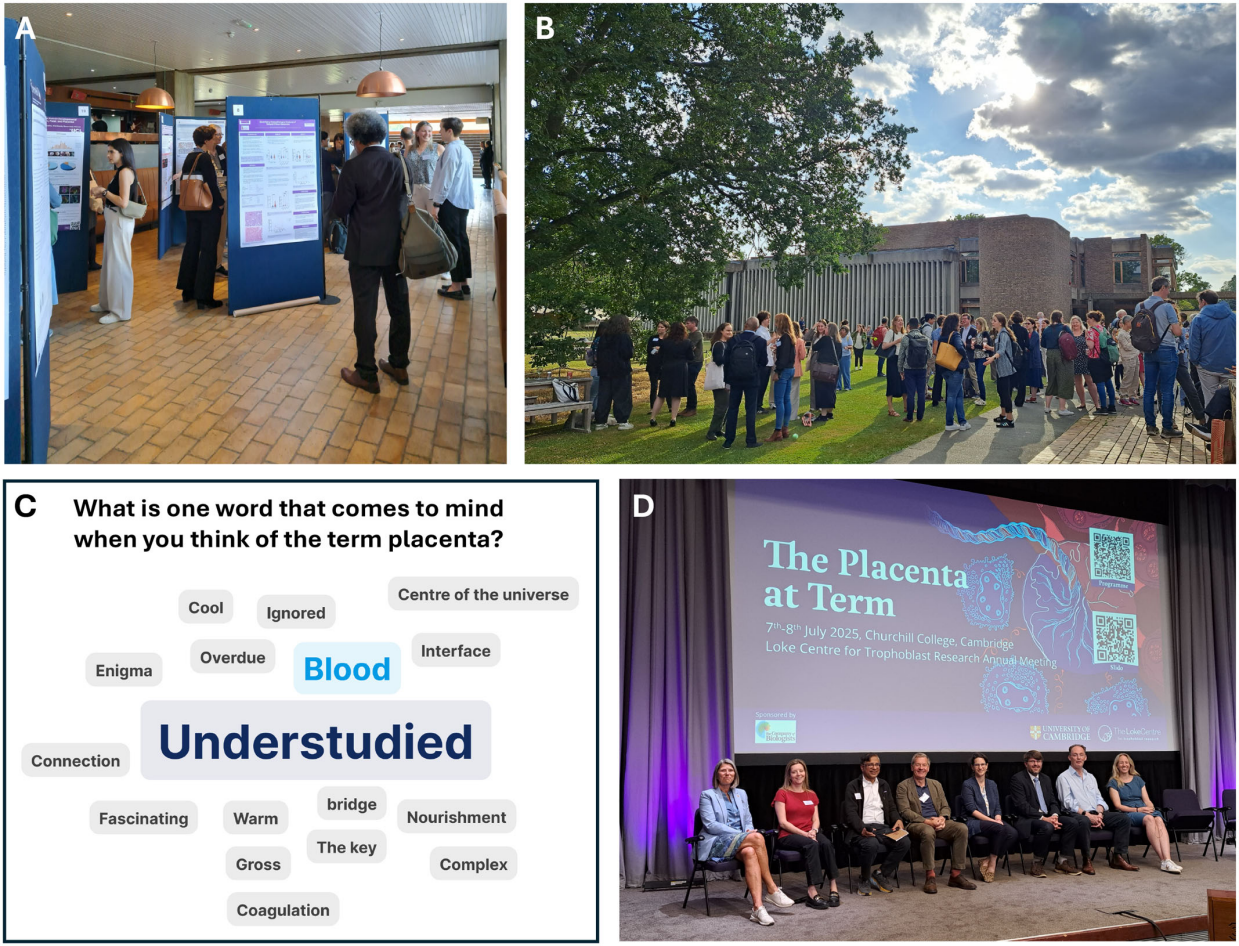
**Community and perspectives at the 2025 Loke CTR Annual Meeting.** Opportunities for interaction included poster sessions (A), informal networking during a drinks reception (B), and interactive online activities (C). The meeting concluded with a panel discussion led by keynote speakers (D), with active participation from the wider audience.

The 2025 Loke CTR Annual Meeting created fertile ground for exchange, offering ECRs opportunities to showcase their work and build networks through poster sessions, flash talks, informal discussions, and interactive activities (Fig. 2A-C). The closing panel, led by keynote speakers and enriched by active audience participation, fostered dialogue across career stages and disciplines, reinforcing the meeting's role as both a scientific forum and a community platform (Fig. 2D).

The panel highlighted several enduring challenges; while histopathological evaluation of term placentas remains valuable, it is constrained by post-delivery artefacts, inter-observer variability, and a shortage of dedicated perinatal pathologists. Underrepresentation of practicing clinicians in placental research is another barrier that restricts bench-to-bedside translation. This was evident at the meeting, where clinicians were well represented among invited speakers but made up fewer than 10% of total attendees. Most importantly, examining the placenta after birth captures only the end result of the underlying pathophysiology. Although this approach may provide predictive value for lifelong health risks, it cannot reveal the mechanisms that gave rise to the obstetrical complication. As a result, progress toward developing tools for early diagnosis and prevention during pregnancy remains limited. Alongside these limitations, the meeting emphasised the growing role of bottom-up approaches in bridging gaps in understanding of pregnancy complications. Just as well-conducted history-taking (anamnesis) leads to the correct diagnosis in ∼80% of cases (Hampton et al., 1975), unbiased high-throughput analyses of patient-derived samples let biology itself guide us to the underlying mechanisms of obstetric complications.

Future directions emphasised the need to move beyond the ‘end-point’ placenta by applying longitudinal functional imaging to study placental function throughout pregnancy. Complementing this, systematic biobanking combined with extensive molecular profiling could transform placental research in much the same way it has revolutionised oncology, enabling deeper biological insight and targeted interventions. Progress will also depend on broadening research to the full maternal-placental-fetal interface, including underexplored compartments such as the decidua, and expanding the temporal scope beyond pregnancy to encompass preconception and postpartum periods. Achieving this vision will require cross-disciplinary integration, bringing together fertility specialists, obstetricians, gynaecologists and basic scientists to investigate these less explored domains.
